# Waterpipe Smoking among Middle and High School Jordanian Students: Patterns and Predictors

**DOI:** 10.3390/ijerph10127068

**Published:** 2013-12-12

**Authors:** Sukaina Alzyoud, Linda S. Weglicki, Khalid A. Kheirallah, Linda Haddad, Khalid A. Alhawamdeh

**Affiliations:** 1 Department of Community and Mental Health, Faculty of Nursing, Hashemite University, Zarqa, P.O. Box 150459, 13115, Jordan; E-Mail: kamh1970@yahoo.com; 2 Wayne State University, College of Nursing, Detroit, MI 48202, USA; E-Mail: weglickils@mail.nih.gov; 3 Department of Public Health, Faculty of Medicine, Jordan University of Science and Technology, Irbid, P.O. Box 3030, 22110, Jordan; E-Mail: kkheiral@gmail.com; 4 School of Nursing & Institute for Drug and Alcohol Studies, Faculty of Nursing, Virginia Commonwealth University, Richmond, VA 23298, USA; E-Mail: lhaddad2@vcu.edu

**Keywords:** Jordan, waterpipe smoking, predictors, youth

## Abstract

Despite the increase in attention to waterpipe tobacco smoking, the patterns and predictors of this method of tobacco use among Jordanian youth are not well known. The current study was conducted to assess the patterns and the predictors of waterpipe tobacco smoking among school aged students in one of Jordan’s Central Governorates. A cross-sectional survey was conducted to investigate the patterns and predictors of waterpipe tobacco smoking among youth (grades 6, 8, 10 and 12). Using a multistage random sampling more than 1,000 students was selected. Data were collected using the Arabic Youth Tobacco Use Composite Measure (YTUCM). Waterpipe smoking was assessed for “past 12 months”, “past month” and “past week”. Students’ ages ranged from 11 to 18 years, (mean age ± 14.7; SD ± 1.9 years). The percentage of girls who smoked waterpipe was greater for all frequencies of use than it was for boys. Age, gender, and belief that smoking makes more friends were predictors of smoking among study participants. This is the first known study to examine waterpipe smoking among youth aged 11 and 12. Our findings illustrate the need for public health campaigns to reach and educate youth, their families, teachers and school systems regarding the growing recognized health risks of waterpipe smoking.

## 1. Introduction

Numerous studies have indicated an alarming increase in waterpipe smoking [1,2,3,4,5]. Waterpipes are known by different names depending on the region of the World. These include, but are not limited to, hookah, narghile, arghile, shisha, and hubble-bubble [6]. In Jordan, waterpipe smoking is commonly known as narghile or arghile. 

Research has established that waterpipe tobacco smoke contains and produces toxic substances similar to those produced by cigarette smoke, including carcinogenic polycyclic aromatic volatile aldehydes [7], hydrocarbons [8], carbon monoxide [9], and nicotine [10,11,12]. Eissenberg and Shihadeh [9] reported that a single waterpipe tobacco smoking session may involve the inhalation of 50 to 100 times the smoke volume inhaled from a single cigarette. Waterpipe smokers who smoke once a day were found to have the same plasma nicotine concentration as cigarette smokers who smoke 10 cigarettes a day [13]. Evidence also suggests that waterpipe smoking is associated with negative health outcomes similar to those of cigarette smoking. Over the years researchers have identified, the association between waterpipe tobacco smoking and lung cancer, respiratory illness, low birth-weight, blood pressure and heart rate increase, and periodontal disease [6,14,15,16,17]. 

Waterpipe smoking has most often been associated with countries in the Middle East and North Africa [MENA], including Egypt, Kuwait, Lebanon, Syria, Iran, United Arab Eremites, Saudi Arabia, Qatar, Oman, and Jordan. Reports of adolescents’ waterpipe smoking were mainly provided through the Global Youth Tobacco Survey [GYTS] which is conducted by Center for Disease Control [CDC] and focuses primarily on prevalence data of predominately adolescents who are 13 to 15 years of age. The GYTS has only reported data on current smoking of waterpipe among adolescents. Accordingly, *current waterpipe* was defined as those who reported having smoked waterpipe during the last 30 days. Research indicates that adolescents from these countries self-reported having “currently tried” waterpipe tobacco smoking ranges from 2 to 35% [18,19,20,21,22,23]. In Oman, it was reported that 1.5 to 27% of adolescents, 13 to 16 years of age, currently smoked waterpipes [22,23,24], while in Lebanon, which have the highest prevalence of current waterpipe smoking, around 35% of Lebanese adolescents 13 to 15 years of age reported currently waterpipe smoking [23]. Among adolescents aged 11 to 18 years of age, however, data regarding waterpipe smoking prevalence is limited in MENA countries, especially Jordan. 

In Jordan, however, the estimated prevalence of having ever smoked any form of tobacco among adolescents was 18% in 1999, 13% in 2004, 16% in 2007 [25] and 26% in 2009 [22], whereas about 21% of Jordanian adolescents aged 13 to 15 currently smoke waterpipes, with higher prevalence rates among males (27%) than females (16%) [22]. Mzayek and colleagues [26] conducted a longitudinal study which followed 1,702 seventh-grade students (13 years of age) in one of Jordan’s northern governorates to assess the patterns in smoking initiation among youth for both cigarettes and waterpipes. They reported that the prevalence of having ever smoked waterpipe increased 1.8 times during the 2-year follow-up period (from 26% to 46%; *p* < 0.01), while current waterpipe smoking increased 1.4 times during the same period (from 13% to 19%; *p* < 0.01) among the study sample.

Waterpipe smoking behavior is being continuously observed in MENA countries. It mostly occurs in a social setting and gatherings such as among family and friends in homes [27,28,29,30], or in places that offer ready-to-smoke waterpipes to customers, such as restaurants and cafes [27,31,32,33]. Other important factors that may be relevant to the rising increase in waterpipe smoking by youth is the misconception that waterpipe smoking is less harmful than smoking cigarettes [34,35,36], that tobacco toxins are filtered via the water reservoir [35,37] and that the tobacco and water reservoir result in less irritation to the mouth and airways as compared to cigarettes [16]. An additional factor resulting in the uptake of waterpipe smoking is that waterpipe smoke has a pleasant odor that is attributed to the growing number of available flavored additives (e.g., lemon mint, white peach, mint chocolate chill) [14]. While cigarette smoking by females continues to be considered a social taboo in conservative Arabic societies [6], waterpipe smoking among females of all ages is considered a socially acceptable behavior. Youth in general, and young girls in particular, may be more willing to smoke waterpipes compared to smoking cigarettes due to its mystic, growing social acceptance, and the misconception that waterpipe smoking is safe or safer than smoking cigarettes [38]. In addition, youth, particularly girls, may favor the positive sensory characteristics—attractive smell and taste of the various fruitful additives—obtained through waterpipe smoking [39]. Another important factor contributing to the growing use of waterpipe smoking by youth is parent/family member use. A study conducted among a convenience sample of Arab-American high school students [5] found that if one or more family members smoked waterpipes in the home, adolescents were 6.3 times more likely to be current waterpipe smokers. Additionally, studies in the MENA region have indicated that adolescents are more likely to smoke waterpipes if they had a parent who smoked waterpipe or cigarettes [24,40]. Several studies indicate that Jordanian adult waterpipe smoking use by adults are high, ranging from 53% to 61% [27,41,42] which may result in parent/family member use modeling of waterpipe smoking behaviors. 

Previous studies in Jordan such as the GYTS and Mzayek *et al.*, [26] had only included youth who are aged 13 to 18 years. Moreover, these studies have mainly reported the prevalence of current tobacco use and did not focus solely on waterpipe smoking. Therefore, the primary purpose of this study was to assess the use of waterpipe tobacco smoking among a random sample of school students, 11 to 18 years of age, who live and attend school in one of the largest governorates of Jordan. The current study will include adolescents from urban, suburban, and semirural areas. Previous studies mainly included youth from urban areas [26,43]. The study also examined predictors (e.g., socio-demographic determinants such as age, gender, and parent and friend use of smoking) and attitudes towards waterpipe smoking by Jordanian youth.

## 2. Methodology

### 2.1. Design

A cross-sectional study was conducted from February to June, 2012 in order to determine the patterns, predictors, and frequency of waterpipe tobacco smoking among high school-aged students in Zarqa, one of the major governorates of central Jordan.

### 2.2. Setting

The study was conducted in the Zarqa Governorate located in the central part of Jordan; east of the Capital Amman. The Governorate has 15% (total population of 931,100) of Jordan’s population [44], and is considered the second largest industrial and third most densely populated governorate in the country. This Governorate was chosen because it represents adolescents from urban, suburban, and rural population densities.

### 2.3. Population

The total number of students enrolled in these schools is estimated to be 203,433; 104,489 enrolled in boys’ schools and 98,944 enrolled in girls’ schools [45]. The three Zarqa districts contain a total of 75 schools that contain basic level grades only (*i.e.*, grades 1–10) and schools that have high level grades (*i.e.*, grades 11–12) which are distributed across the governorate’s three districts. However, for the purpose of this study only school that include 6^th^ grade and higher were considered for participant recruitment, as the researchers only aim to include six graders and higher as study participant. Zarqa’s three Educational Districts include basic education level (grades 1 to 10) and high school education level (grades 11 to12). Students in Zarka schools are segregated in schools based on gender; boys’ and girls’ schools. A power analysis was conducted to determine the required study sample size. The minimum sample size required for a two sided significance level of 95%, a power of 80%, two equal groups, with prevalence of tobacco use among males and females estimated to be 27% and 16%, respectively as provided by the GYTS data set of 2009, was estimated to be a total of 680 subjects.

### 2.4. Recruitment, Permission, and Data Collection

The study protocol was approved by the Institutional Review Board of the Hashemite University (HU) and Zarqa Governorate Educational District. The current study only included governmental schools. Verbal permission was also obtained from each school principle prior to data collection. Informed consent was obtained from all participants before completing study measures. Subjects were selected using a multistage cluster random sampling technique. First, a list of schools was requested from the three Educational Districts. Two lists were provided from each district; one for boys’ schools and one for girls’ schools. Schools within each list did not follow any specific order. Each list contained both schools in basic and high school educational levels. Schools varied between middle grades only, high school grades only, and mixed schools of middle and high school grades. Schools were randomly selected from each list using systematic random sampling technique where every 5th school was randomly selected by the research assistant. The needed number of schools to achieve the desired sample size was 11 schools, which were selected at this stage. In the second stage, classrooms from each selected school were randomly selected. Using this sampling strategy, a total of 1,050 school students were invited to participate in the study, of which 95% completed the questionnaires.

### 2.5. Measures

The Arabic Youth Tobacco Use Composite Measure (YTUCM), developed originally in English and translated into Arabic, is a 136-item composite measure that designed as a single measure to assess known predictors of tobacco use by youth [46]. For the purpose of this study, the researchers used select items, standardized questions, of the YTUCM. These standardized questions were: (1) waterpipe tobacco use experience (current smoking status, age of first smoking, usual places of waterpipe smoking), (2) waterpipe tobacco smoking experience categories: past month use (defined as use of waterpipein the past 30 days, even if a single puff), past week smoking (smoking of waterpipein the past 7 days, even if a single puff), and past year smoking (smoking of waterpipe in the past 12 months, even if a single puff), (3) hours of exposure to waterpipe secondhand smoke, (4) tobacco exposure including knowledge and attitudes, (5) social situations: family waterpipe tobacco use patterns, school factors, academic performance, peer waterpipe smoking behavior, offers of waterpipe smoking, and health perception. Health perception variable was measured using two items: the first one assessed whether participants think it is safe for their health to smoke tobacco for only a year or two as long as they would quit after that time frame. The second item required participants to rate their health on a scale from 0 to 5, for 0: represents not at all healthy; 1: very unhealthy; 2: somewhat unhealthy; 3: healthy; 4: very healthy; 5: most healthy ever. Response options in the Measure vary based on the construct and items measuring that construct. They range from Likert-type responses; yes/no responses, fill in the blank, to ranking using a number line. Participants were also asked about their demographic and background information. Of this information they were asked about their paternal occupation to reflect their Socio-Economic Status SES. This item was measured by asking students “What is your Dad’s usual job?”, based on their answers a new variable was created to categorize paternal occupation into three categories *blue collar* such as vocational workers, *white collar—*like teachers, and *unemployed*. 

### 2.6. Data Analysis

The Statistical Package for the Social Science (SPSS) version 17 was used for data management and analysis. Univariate, bivariate and multivariate analyses were performed. Frequency distribution and means were reported along with the adjusted Odds Ratios (OR) and 95% Confidence Intervals C.I. (OR: 95% C.I.). Backward elimination multiple logistic regression was performed to find the set of best predictors of 30-day waterpipe smoking. *P*-values < 0.05 were considered significant. The collinearity was checked for in the logistic regression model. 

## 3. Results

A total of 1,050 questionnaires were distributed; response rate was 95.2%. Students’ age ranged from 11 to 17 years, (mean ± SD = 14.7 ± 1.9 years). About 54% of participants were females (n = 459), and the majority were Jordanians (97%). About 8% of all participants smoked waterpipes during social gatherings, while 44% of the participants had friends who also smoked waterpipes. See Table 1 for participants’ other characteristics. 

**Table 1:** Sample demographic characteristics (n = 993).

Variable	Number	Percent *
Age in years		
11–12	189	19
13–14	221	22
15–16	397	40
17–18	186	19
Gender		
Male	459	46
Female	534	54
Grade		
6th	189	19
8th	221	22
10th	397	40
12th	186	19
Paternal Occupation		
Blue collar	359	36
White collar	485	49
Unemployed	177	18
Number of siblings		
Equal to or less than 6 siblings	521	53
More than 6 siblings	441	44
Having one or more of their five closest friends who use waterpipe?		
Yes	435	44
No	558	56
Usual place of waterpipe tobacco smoking		
Non Smoker	718	72
Home	16	2
Café	47	5
Friend’s houses	21	2
Social gatherings	83	8
Public places (e.g., parks, street corners etc)	4	0.4
Restaurants	61	6
Number of smoked waterpipe heads per day		
Less than 1 head	78	23
1 head	91	27
2 heads	62	19
3 or more heads	48	14

### 3.1. Pattern of Waterpipe Smoking

Thirty six percent (n = 357) of youth have tried waterpipe smoking regardless of experience categories, 36% of them were male while 64% of them were female. Of the youth, 36% (n = 357) smoked water pipe at least once in the past year, 34% (n = 334) smoked waterpipes at least once in the past month, and 30% (n = 299) smoked in the past week (Table 2). Waterpipe smoking was more prevalent among girls than boys. Differences in prevalence rate between males and females were statistically significant.

Mean age of first waterpipe smoking was 13 years (SD ± 1.95 years). The results indicated that youth started waterpipe smoking as young as 11 years of age (Table 3). Youth who were current waterpipe smokers reported social gatherings (23% of waterpipe smokers n = 83 of 357) as the most common place for waterpipe use. Students mostly smoked one head (27%) or less (23%) per day. Offers to smoke waterpipe were mainly made by friends (60%) or by family members (53%) Table 1. Participants were also asked about their perception of health, and beliefs about waterpipe smoking harms (see Table 4).

**Table 2:** Distribution of study participants by waterpipe smoking and gender (n = 993).

Waterpipe Ever Use Frequency	Total		Male		Female	
	Yes	No	Yes	No	Yes	No
Past year	357	633	127	332	230	301
	*36%*	*64%*	*28%*	*72%*	*43%*	*57%*
Past month	334	658	112	347	222	311
	*34%*	*66%*	*24%*	*76%*	*42%*	*58%*
Past week	299	690	99	358	200	332
	*30%*	*70%*	*22%*	*78%*	*38%*	*62%*

**Table 3:** Distribution of study participants by waterpipe smoking and age (n = 993).

Age	Past year		Past month		Past week	
	Yes	No	Yes	No	Yes	No
11 to 12 years	35	154	30	159	25	164
	4%	*16%*	*3%*	*16%*	*3%*	*17%*
13 to 14 years	66	155	59	162	52	167
	*7%*	*16%*	*6%*	*16%*	*5%*	*17%*
15 to 18 years	256	324	245	337	222	359
	*26%*	*33%*	*25%*	*35%*	*22%*	*36%*
Total	357	633	334	658	299	690
	*36%*	*64%*	*34%*	*66%*	*30%*	*69%*

Participants were asked to rate their health on a scale ranging from 0 to 5, (not at all healthy to most healthy ever). After the initial analysis the scale was recorded and redistributed into four categories. Due to low percentage of youth who rated their health for *not at all healthy* and *very unhealthy* categories they were collapsed into one category which is *not at all healthy*. Therefore, resulting in four categories (most healthy ever, very healthy, healthy, and not at all health that were included in the final analysis (as shown in Table 4). The proportion of students who perceived their health as “not at all healthy” was higher among waterpipe users compared to none users. For none waterpipe users, the proportion of students who perceived their health as “most healthy ever” was higher. For example, 32%, 32.6% and 34% of past year, past month and past week waterpipe users, respectively, perceived their health as “not at all healthy” compared to 23.2%, 23.5% and 23.5% of none users, respectively. When asked about their beliefs that smoking waterpipes for a period of time will not harm their health, waterpipe using participants were almost split in half (51% to 49%) in their beliefs regarding whether or not it would harm their health.

**Table 4:** Distribution of waterpipe frequency use and participants’ perception of health.

Health Scale	Past year		Past month		Past week	
	Yes	No	Yes	No	Yes	No
	No.	No.	No.	No.	No.	No.
	*(%)*	*(%)*	*(%)*	*(%)*	*(%)*	*(%)*
Not at all healthy	112	144	106	152	99	159
	*32%*	*23%*	*33%*	*24%*	*34%*	*24%*
Healthy	119	170	110	179	102	187
	*34%*	*27%*	*34%*	*28%*	*35%*	*28%*
Very healthy	69	139	63	145	59	148
	*20%*	*22%*	*19%*	*22%*	*20%*	*22%*
Most healthy ever	49	167	46	170	32	183
	*14%*	*27%*	*14%*	*26%*	*11%*	*27%*
Total	349	620	325	646	292	677
	*100%*	*100%*	*100%*	*100%*	*100%*	*100%*

### 3.2. Predictors of Waterpipe Smoking

The logistic model was performed to examine the relationship between the outcome variables current smoker with participants’ socio-demographics, perception of health, family and peer waterpipe smoking, beliefs about waterpipe smoking harms, and number of offers to smoke waterpipes by friends and family (see Table 5). 

For each one year increase in age, the odds of being a 30-day waterpipe smoker significantly increased by 1.6 times (95% Confidence Interval [C.I.]: 1.02–2.41). Girls were about two times more likely to be past 30-day waterpipe smokers compared to boys (Odd Ratio [OR], 95% C.I.: 1.48, 1.01–2.18). Participating in extracurricular activities twice a week or more significantly increased the odds of being a past 30-day waterpipe smoker by 1.66 times compared to those who do not participate in such activities (95% C.I.: 1.10–2.52). Compared to students who reported their school achievement to be excellent, students who reported good or poor achievement were 1.73 (95% C.I.: 1.03–2.90) and 1.88 (95% C.I.: 1.02–3.36) times, respectively, more likely to be past 30-day waterpipe smokers. Participants who reported that smoking waterpipes helps them make more friends were significantly about three times as likely to be past 30-day waterpipe smokers as those who did not hold such a perception (OR, 95% C.I.: 2.80, 1.89–6.67). Family discussion regarding the danger of smoking significantly reduced the likelihood of being a past 30-day waterpipe smoker; (OR, 95% C.I.: 0.5, 0.44–0.74). 

**Table 5:** Adjusted effect of predictors of waterpipe smoking.

Variable	OR	95% C.I.
Age (in years)	1.57	1.02–2.41
Gender (Female)	1.48	1.01–2.18
Participating in extracurricular activities		
Do not participate	Ref	..
Once per week	1.16	0.70–1.91
Twice or more per week	1.66	1.10–2.52
School Achievement		
Excellent	Ref	..
V. good	1.20	0.70–2.03
Good	1.73	1.03–2.90
Poor	1.88	1.01–3.36
I think smoking waterpipe helps me make friends	2.80	1.89–6.67
Has anyone in your family discussed the dangers of smoking with you?		
Yes	0.5	0.44–0.74
I think it is safe to smoke waterpipe for only a year or two as long as I quit after that	1.57	1.10–2.27
Number of closest five friends who smoke waterpipes	1.43	1.30–1.58
Number of offers of waterpipe smoking received from friends in the past 30 days	1.07	1.01–1.14
Number of offers of waterpipe smoking received from family in the past 30 days	1.23	1.12–1.35

Youth who believed that it is safe to smoke waterpipes for only a year or two as long as one can quit after that were about two times as likely to be past 30-day waterpipe smokers as compared to those who did not believe so (OR, 95% C.I.: 1.57, 1.10–2.27). As the number of smokers among the five closest friends increased by one friend, the probability of being a past 30-day smoker significantly increased by 1.4 times (OR, 95% C.I.: 1.43, 1.30–1.58). As the number of waterpipe smoking offers by friends and family increased by one offer, the likelihood of being a past 30-day waterpipe smoker significantly increased by 1.1 (95% C.I.: 1.10–1.14) and 1.2 (95% C.I.: 1.12–1.35) times, respectively. 

## 4. Discussion

This study presents the pattern of waterpipe smoking among middle and high school students in Jordan. Our study is among the first studies in Jordan which investigated this growing form of tobacco use among youth. A random sample of middle and high school students from a major governorate in Jordan was recruited for the study; we found that students self-report of waterpipe smoking ranged from 30% for the past week, 34% for the past month, and to 36% for the past year.

An interesting finding was that approximately 3% (n = 32) of 11 to 12 year olds reported have smoked waterpipes. As few studies from the Middle East and North Africa, where waterpipe smoking has a long history of use, have looked at waterpipe smoking at this very young age, it calls attention to the need to begin “say no to youth tobacco use programs” and to include attention to waterpipe smoking [5,47]. A significant finding of this study is that for each one year increase in age, the odds of being a 30-day waterpipe smoker significantly increased by 1.6 times. This indicates that Jordanian youth in this governorate are more likely to report “current waterpipe smoking” as they age. This was consistent with findings in previous studies in the MENA region [33]; our results support previously reported studies noting that as youth age, they are more likely to engage in waterpipe tobacco use [5]. Such a finding raises concerns regarding the health problem of waterpipe smoking among adolescents, and they emphasize the need to address the factors that cause waterpipe smoking among adolescents. Furthermore, the study findings indicated that girls were more likely to be waterpipe smokers than boys. This finding is inconsistent with previous studies in Jordan and the region where most of the studies indicated that waterpipe is more prevalent among boys [24,33,48]. However, Mzayek and colleagues [26] reported that the pattern of waterpipe smoking among Jordanian school students is the same among boys and girls. The current study finding could be explained by the increasing trend of waterpipe smoking among youth including females that was addressed by several researchers [6,38]. Moreover, our results are consistent with previous work in which females would feel encouraged to smoke waterpipe because it is socially acceptable and has positive sensory characteristics such as the attractive smell and taste [39].

Students reported being offered to smoke waterpipes by friends and family members. This result is also consistent with previous studies where adolescents used waterpipes at home or at social gatherings among friends or family members [27,33,49]. Our findings indicate that students were more likely to use waterpipes when they received offers from friends and family members. This could be contributed to family members’ belief that waterpipe smoking is less harmful than cigarettes [41,50] and to the social acceptability of waterpipe smoking among adults [6,49]. Youth may feel the desire try smoking waterpipe when offered by friends for a number of reasons. These include perceiving that their friends look cool when smoking waterpipes [50], viewing waterpipe smoking as the trendy habit among friends [1,30,51], sensing that they belong to their friends, and peer pressure [49]. These factors support our finding that having friends who smoked waterpipes and the belief that it helped them to make friends predicted students’ waterpipe use; which suggests that interventions aimed at reducing waterpipe smoking need to consider adolescents’ close social network such as friends and family members. Our findings also suggest that waterpipe smoking has become a socially acceptable practice among the study population. 

Students in the current study were more likely to use waterpipes when they believed that it would not harm their health within one or two years of use. This is consistent with studies where adolescents tend to smoke waterpipes believing that it will not harm their health. As indicated previously youth reported that they were offered waterpipes by their parent, which may attributed to their perception that waterpipe smoking is not harmful. However, it has been documented that waterpipe smoking has the same addictive and harmful effects to health as cigarettes [9,14,15,50]. Furthermore, this finding suggests that waterpipe smokers may underestimate the risks to their own health. This finding is very important and can be used in prevention programs which should emphasize that waterpipe smoking can be addictive and harmful even if it is used for a short period of time. An essential finding when developing prevention interventions for tobacco use among youth is that students believe that using waterpipes will result in poor health. Including parents should be considered when developing prevention intervention programs that target tobacco use among youth. This finding provides an important aspect to health care professionals and decision makers when planning and implementing public health interventions.

## 5. Conclusions

In conclusion, the pattern of Jordanian adolescent waterpipe smoking is similar to their counterparts in the region and in developed countries. In addition, waterpipe smoking among youth is increasing and is now almost as common as cigarette smoking. Youth in this study were more likely to use waterpipes when they perceived it as harmless to their health. This emphasizes the lack of awareness regarding the negative consequences of waterpipe smoking among the young and perhaps their families. In addition, our findings illustrate the need for public health campaigns to reach and educate youth, their families, teachers and school systems regarding the growing recognized health risks of waterpipe smoking.

### 5.1. Study Limitations

Despite several strengths, including a large random sample and a high response rate, limitations should be noted. First, recruitment from one governorate may limit the generalizability of our findings across Jordan; however, the study sample characteristics and distribution is equivalent to the country’s youth characteristics. Second, reported use of waterpipe was based solely on self-reporting. Another limitation could be the inclusion of variables that were uncontrolled for such as socioeconomic status. 

### 5.2. Implications and Future Research

As youth start waterpipe smoking at an early age, thinking waterpipes will not hurt their health, smoke it as a social event, and it is becoming more popular among girls, prevention and intervention efforts should (1) focus on the social context and norm perception of smoking and (2) the role of peers and friends in prevention. In addition to the need to examine waterpipe smoking by youth who are very young (perhaps 10 to 12 years of age), studies are needed to examine waterpipe smoking by Jordanian and Middle Eastern and North African college-age young adults As waterpipe smoking increases with age in youth, understanding the social context related to waterpipe use, including the impact of social networking and technology (e.g., Twitter, Facebook, *etc.*) on it use and trajectory of tobacco use patterns in this population is important in designing future programs to decrease the growing uptake and curtail the lifelong use of tobacco in any form.
